# Supplementary figures and images for: Tissue-specific activities of the Fat1 cadherin cooperate to control neuromuscular morphogenesis
Source: PLoS Biol. 2018 May 16;16(5):e2004734. doi: 10.1371/journal.pbio.2004734 (PMC5973635; doi:10.1371/journal.pbio.2004734)

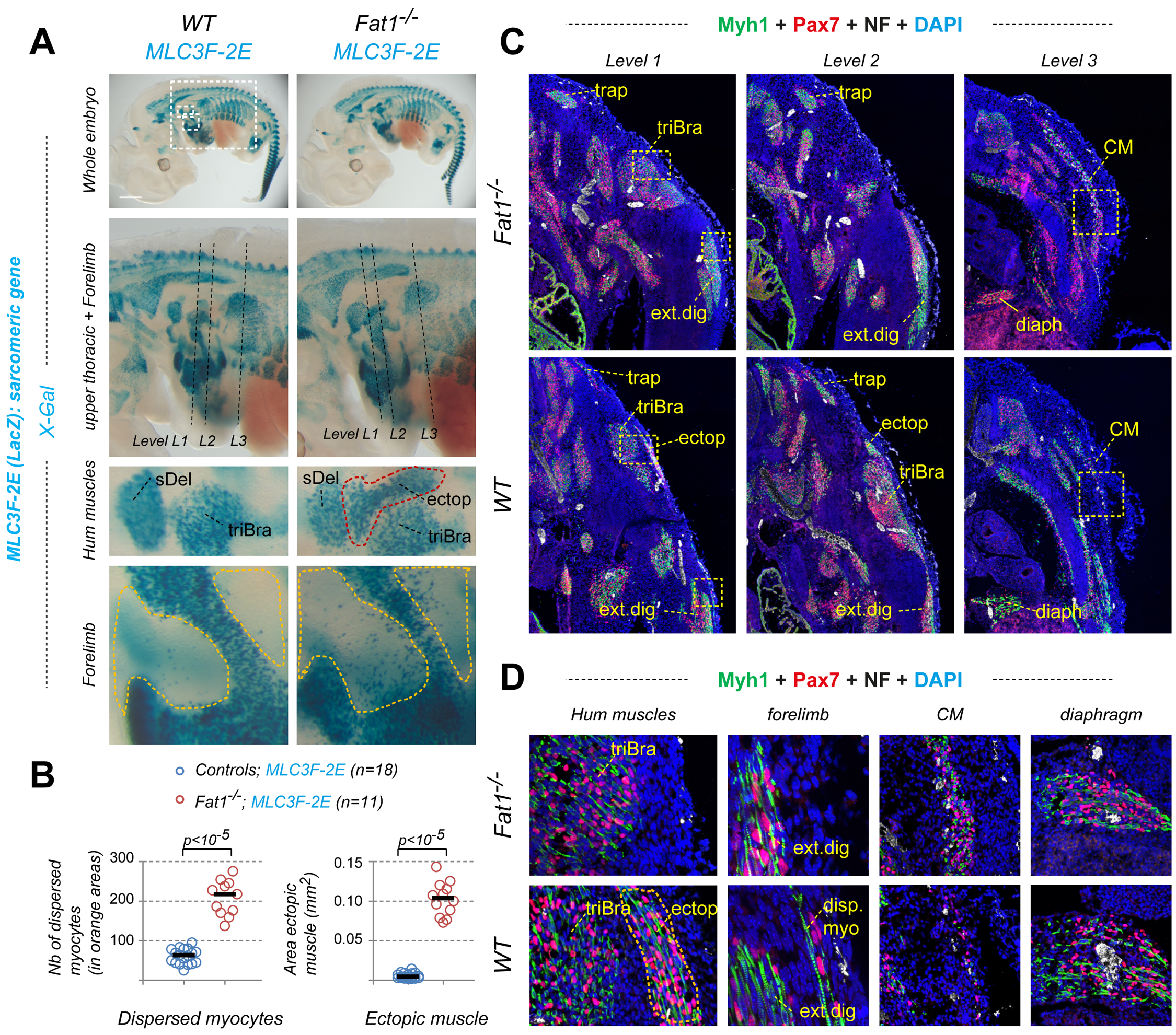

Supplement: S1 Fig — (A) Whole-mount β-galactosidase staining was performed on Fat1+/+; (left) and Fat1-/- (right) embryos carrying the MLC3F-2E transgene (S1 Table). Top images represent a side view of entire embryos and indicate the position of higher magnification areas, including that of the top pictures in Fig 1A and 1B as well as the upper forelimb region (with scapulohumeral muscles) and distal forelimb, showing a region where dispersed myocytes accumulate in Fat1-/- embryos. The second row images highlight a closer view of the upper thoracic and forelimb region. The dotted lines indicate for each genotype the levels and angle corresponding to the three consecutive sections shown in (C) and (D). (B) Quantification of the number of dispersed myocytes found in orange areas in the forelimb (left plot) and of the area occupied by the ectopic humeral muscle (ectop) appearing between the spinodeltoid (Del) and the triceps brachii (TriBra) muscles (right plot). These data reproduce and confirm our own previous results. Underlying data are provided in S1 Data. (C, D) Cross sections of control Fat1+/- and mutant Fat1-/- E12.5 embryos, featuring three consecutive sections at forelimb levels (Level 1 and Level 2) and upper thoracic level (Level 3), immunostained with antibodies against Pax7 (red), Myh1 (green), and neurofilament (white) and with DAPI (blue). Images in (D) represent high-magnification views of the area highlighted with the yellow dotted square in (C). These data confirm (1) the severe reduction in thickness of the CM muscle (Level 3, and higher magnification in [D]), (2) the presence of a robust ectopic muscle next to the triceps brachii (Levels 2 + 3, and higher magnification in [D]), and (3) the presence of dispersed myogenic progenitors and muscle fibers in the ectopic subcutaneous position in the forelimb (the image in [D] shows higher magnification of an area between the digit extensors and the skin). Lack of obvious phenotype in the diaphragm is also shown. CM, cutaneo [file pbio.2004734.s004.tif]

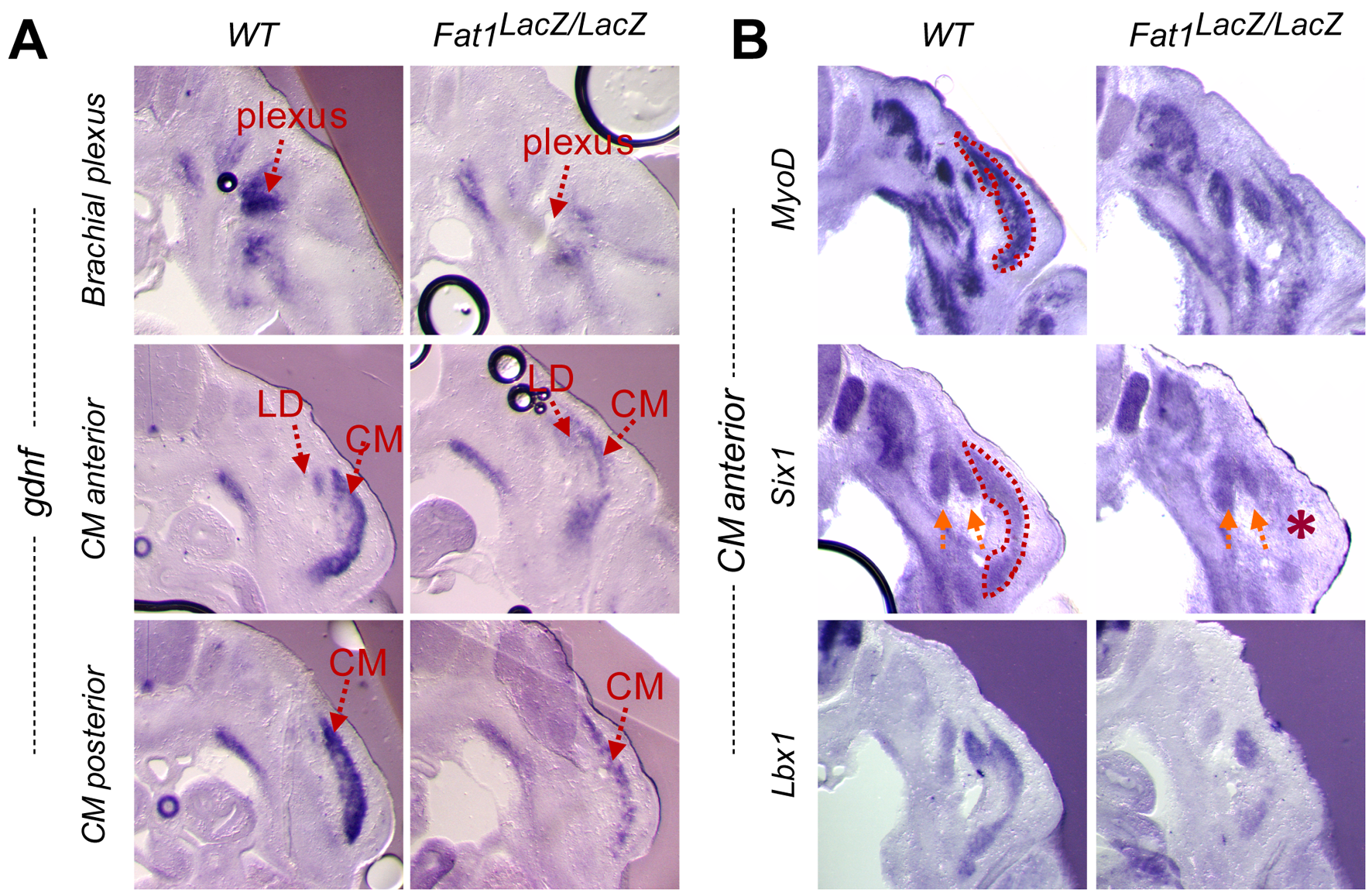

Supplement: S2 Fig — Expression of Gdnf (A) and of three markers of developing muscles (MyoD, Six1, and Lbx1 [B]) was analyzed by in situ hybridization on floating vibratome sections of E12.5 wild-type (left panels) and Fat1LacZ/LacZ embryos (right panels). (A) Gdnf expression is visualized at three successive anteroposterior levels, showing a hot spot at the brachial plexus (mesenchymal cells around passing nerves), where Gdnf expression is drastically reduced by the absence of Fat1, and two successive levels in the CM and LD muscles, where Fat1LacZ/LacZ embryos exhibit a thinner CM with less overall Gdnf signal. (B) On sections corresponding to the anterior part of the CM muscle, expression of markers of muscle differentiations (MyoD, Six1, and Lbx1) also shows that Fat1LacZ/LacZ embryos exhibit a selective loss of staining in the CM and not other neighboring muscle masses. CM, cutaneous maximus; Gdnf, glial cell line-derived neurotrophic factor; Lbx1, ladybird homeobox 1; LD, latissimus dorsi; MyoD, myogenic differentiation 1; Six1, sine oculis-related homeobox 1. (TIF) [file pbio.2004734.s005.tif]

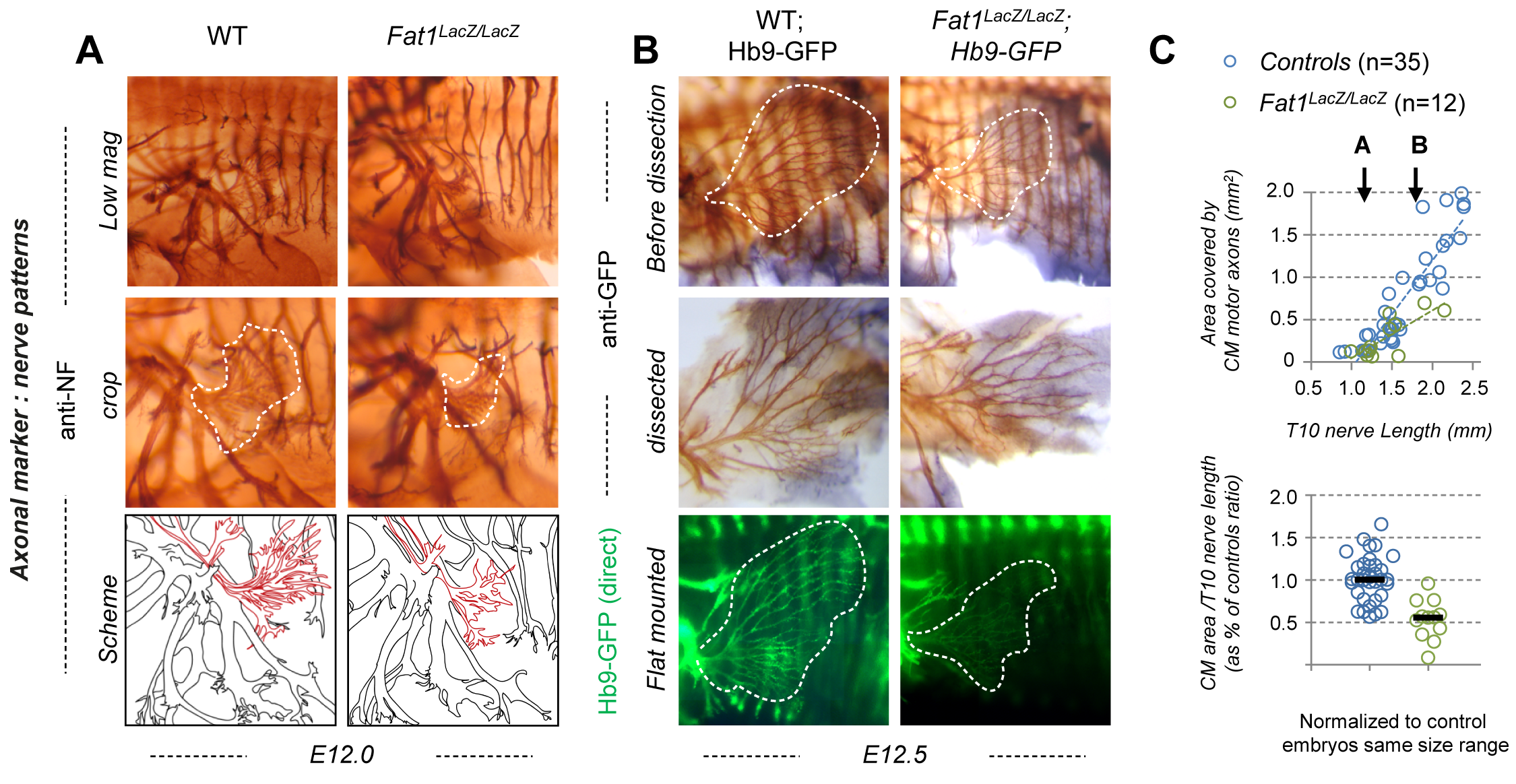

Supplement: S3 Fig — (A, B) The nerve pattern was analyzed by IHC with antibodies against neurofilament (2H3 antibody) (A) or by taking advantage of the Hb9-GFP transgene (S1 Table) (B), which labels motor neurons and their axons. (A) Anti-neurofilament histochemistry on whole-mount wild-type and Fat1LacZ/LacZ embryos at E12.0. (B) Hb9-GFP was visualized with antibodies against GFP (top and middle images) or by direct fluorescence imaging in Fat1+/+; Hb9-GFP+ and Fat1LacZ/LacZ; Hb9-GFP+ embryos at E12.5. For IHC, embryos were cut in half, cleared in BB-BA, and flat-mounted. Upper panels (A, B) are low-magnification images of the embryo flank. Lower panels show high-magnification views of the area containing the CM muscle. The area covered by CM-innervated axons is highlighted in white (middle panels). In the middle panels in (B), axons of vertically oriented thoracic spinal nerves have been manually removed by dissection to improve visibility of CM axons. In lower panels in (B), direct GFP imaging was done on PFA fixed embryos, after flat-mounting of the flank. (C) Quantifications of the relative expansion of the area covered by CM-innervating axons. Upper plot: for each embryo side, the area covered by CM-innervating axons is plotted relative to the length of a thoracic nerve (T10, from dorsal root origin to ventral tip). Arrows point the stages of representative examples shown in (A) and (B). Bottom plot: for each embryo, the CM-innervated area/T10 length was normalized to the median ratio of control embryos, by size range. Blue dots: Fat1+/+ (n = 35, same sample set as in controls of Fig 2); red dots: Fat1LacZ/LacZ (n = 12). Underlying data are provided in S1 Data. BB-BA, benzyl-benzoate/benzyl-alcohol mix; CM, cutaneous maximus; IHC, immunohistochemistry; PFA, paraformaldehyde. (TIF) [file pbio.2004734.s006.tif]

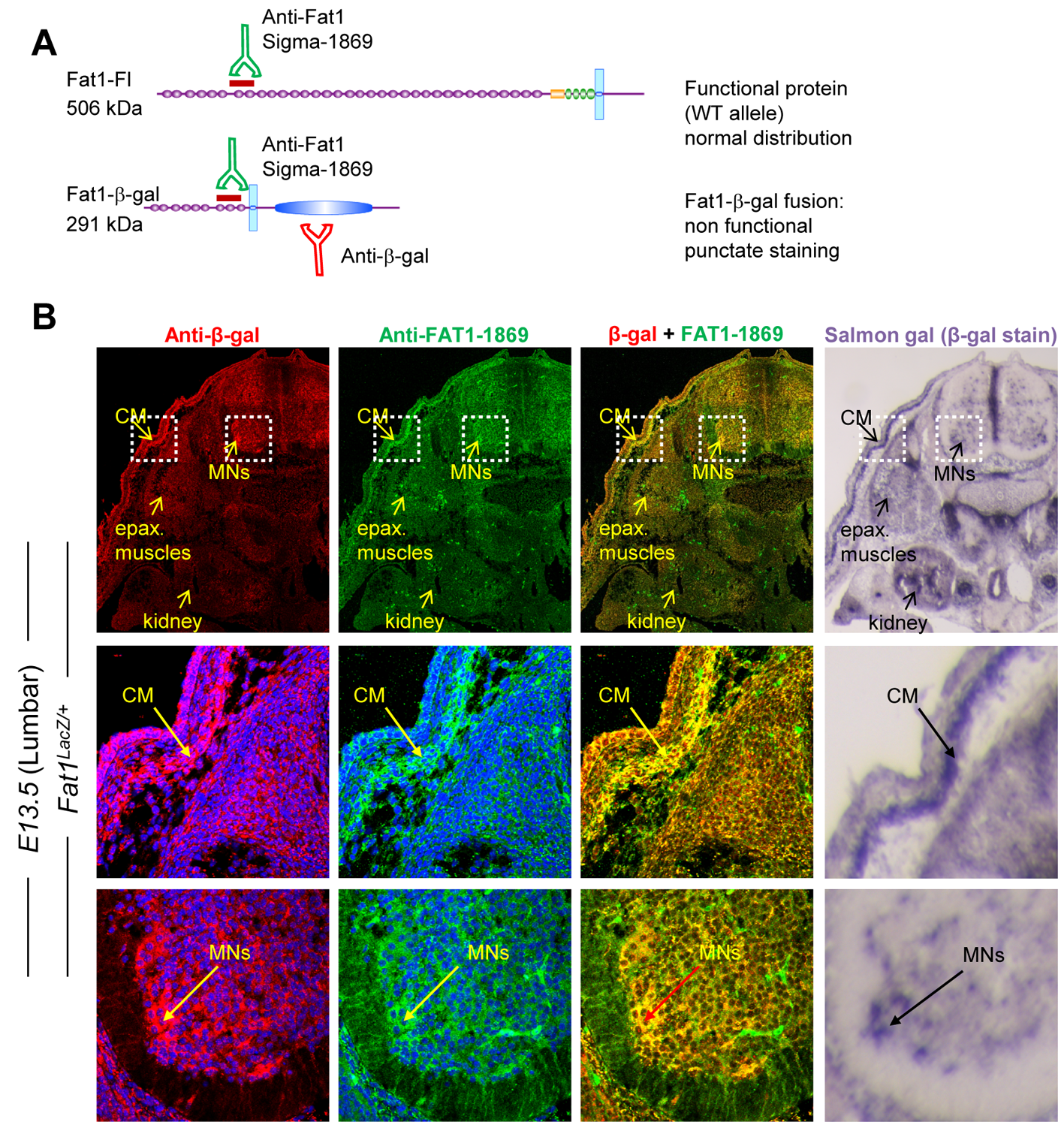

Supplement: S4 Fig — (A) Scheme of the protein products of a wild-type Fat1 allele (full-length Fat1) and of a Fat1LacZ allele (producing a chimaeric protein with the first 8 cadherin domains of Fat1 extracellular domain, fused to an exogenous transmembrane domain in frame with β-galactosidase as intracellular domain). An antibody to Fat1 (Sigma 1869) directed against a portion of the common segment of Fat1 extracellular domain recognizes both proteins, whereas an antibody to β-galactosidase recognizes only the Fat1–β-gal fusion protein, most of which is sequestered in the Golgi apparatus and not localized at the cell membrane. (B) Comparison of immunohistochemical detection of Fat1 in a Fat1LacZ/+ embryo using the anti-β-galactosidase antibody (red), the Fat1-1869 antibody (green), and the pattern of β-galactosidase activity revealed by Salmon-Gal staining on cross sections of an E13.5 mouse embryo at lumbar levels where it is possible to detect both the expression in subsets of MNs and in the caudal-most part of the mesenchymal subcutaneous layer, towards which the CM extends. The Fat1–β-gal fusion protein is mainly detected by both antibodies as punctae in the Golgi, most likely because the protein is misfolded and does not reach the cell membrane. The two stainings perfectly overlap (except for some weak staining of blood vessels in the green channel, mostly due to the secondary antibody) and correspond to the signal detected by Salmon-Gal staining. β-gal, β-galactosidase; CM, cutaneous maximus; epax, epaxial; MNs, motor neurons. (TIF) [file pbio.2004734.s007.tif]

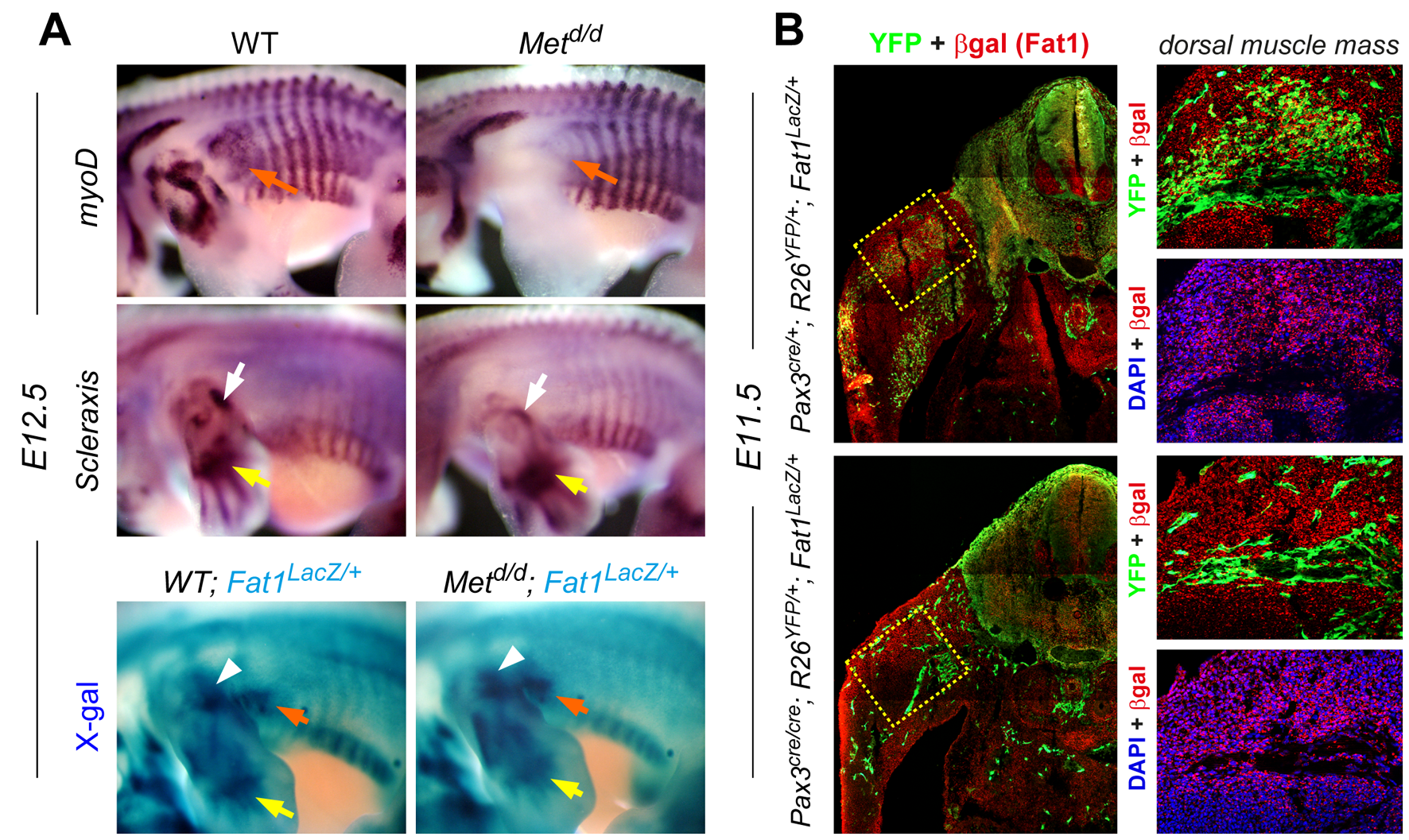

Supplement: S5 Fig — (A) Analysis of MyoD and Scleraxis expression in Fat1LacZ/+ and Metd/d E12.5 embryos, and of Fat1LacZ expression in Fat1LacZ/+ and Metd/d; Fat1LacZ/+ E12.5 embryos was performed by whole-mount in situ hybridization and X-gal staining, respectively. Peripheral sites of Fat1LacZ and Scleraxis expression are maintained in spite of the absence of migratory appendicular muscles, indicating that expression of both genes occurs in non-myogenic/mesenchymal cells. Orange arrowheads indicate the position of the CM muscle; white arrowheads indicate the mesenchyme hot spot in the humeral region of the forelimb; and yellow arrowheads indicate positions of Scleraxis-positive tendons in the distal limb (autopod). (B) Comparison of β-gal (red) and YFP (green) IHC in Pax3cre/+; R26YFP/+; Fat1LacZ/+ (top) and Pax3cre/cre; R26YFP/+; Fat1LacZ/+ (bottom) E11.5 embryos on transverse sections at forelimb levels. The region indicated with a dotted area is shown in higher magnification on the right panels. CM, cutaneous maximus; MyoD, myogenic differentiation 1; Pax3, Paired box 3; R26, Rosa26 Locus; X-gal, substrate for β-galactosidase activity; YFP, yellow fluorescent protein. (TIF) [file pbio.2004734.s008.tif]

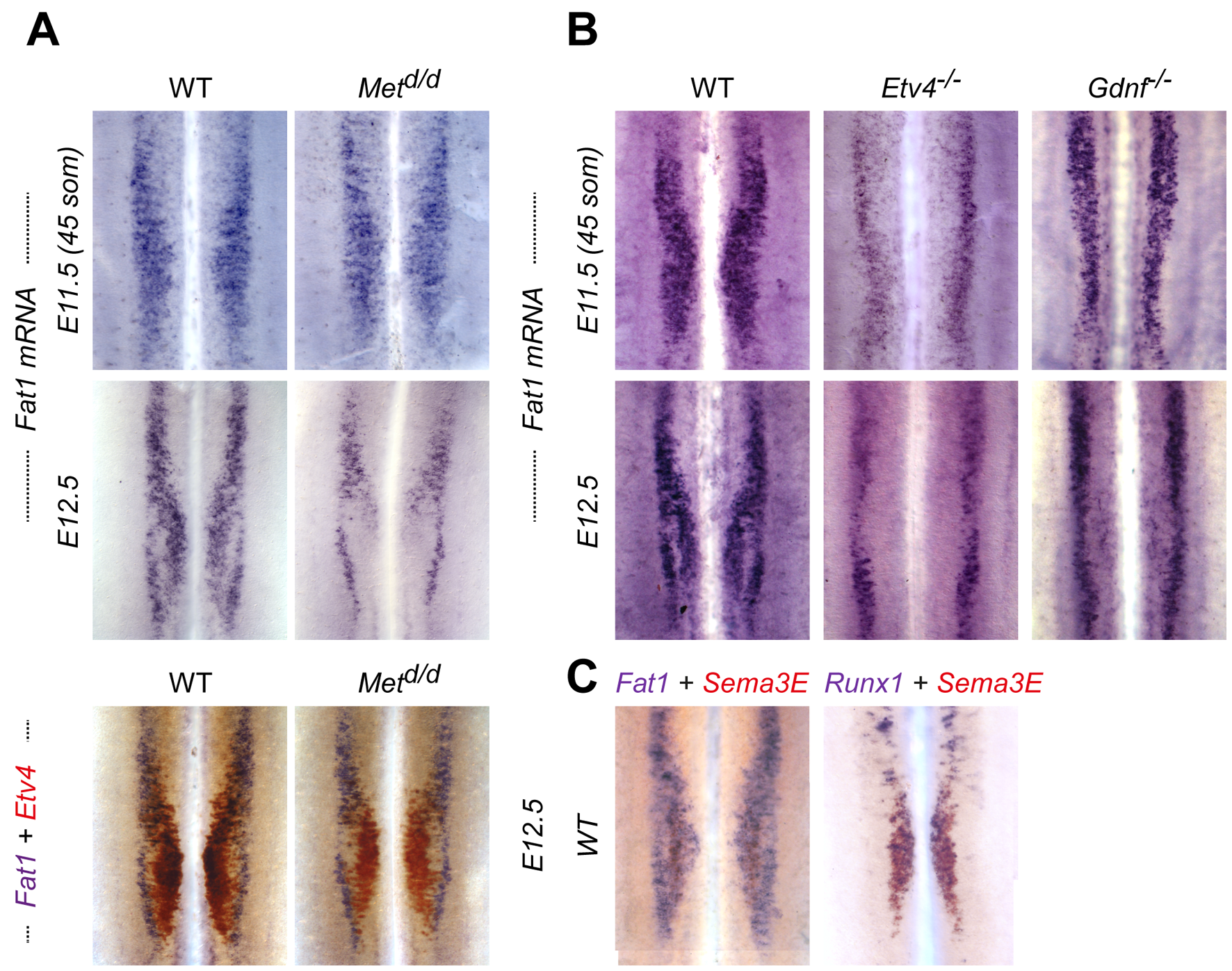

Supplement: S6 Fig — (A) Double ISH analysis of Fat1 (purple) is combined with Etv4 (red) expression in WT and Metd/d E12.5 spinal cords in lower panels. (B) ISH analysis of Fat1 in WT, Etv4-/-, and Gdnf-/- spinal cords at E11.5 (upper panels) and E12.5 (lower panels). (A, B) Onset of Fat1 expression occurs independently of Met (A), of Etv4 and of Gdnf (B) functions and is preserved in Metd/d, Etv4-/-, and Gdnf-/- embryos at E11.5. In contrast, analysis of Fat1 expression at E12.5 reveals alterations consistent with (1) defective maintenance of Fat1 expression in the CM pool in Metd/d spinal cords (A), possibly due to the lack of migrating muscles and subsequent growth factor depletion, and (2) aberrant positioning of the CM pool in Pea3-/- and Gdnf-/- spinal cords (B). (C) Comparison of Fat1, Sema3E, and Runx1 expression in the brachial spinal cord at E12.5 was performed by double ISH with Fat1 (purple) and Sema3E (red) on the left, and with Runx1 (purple) and Sema3E (red) on the right. (D) Fat1 expression in the mouse thoracic spinal cord is shown X-gal staining on sections of Fat1LacZ/+ spinal cords at E12.5, showing expression in all neural progenitors in the ventricular zone, and lack of high- level expression in thoracic motor columns. CM, cutaneous maximus; Etv4, Ets variant gene 4; ISH, in situ hybridization; Runx1, runt related transcription factor 1; Sema3E, semaphoring 3E; WT, wild-type. (TIF) [file pbio.2004734.s009.tif]

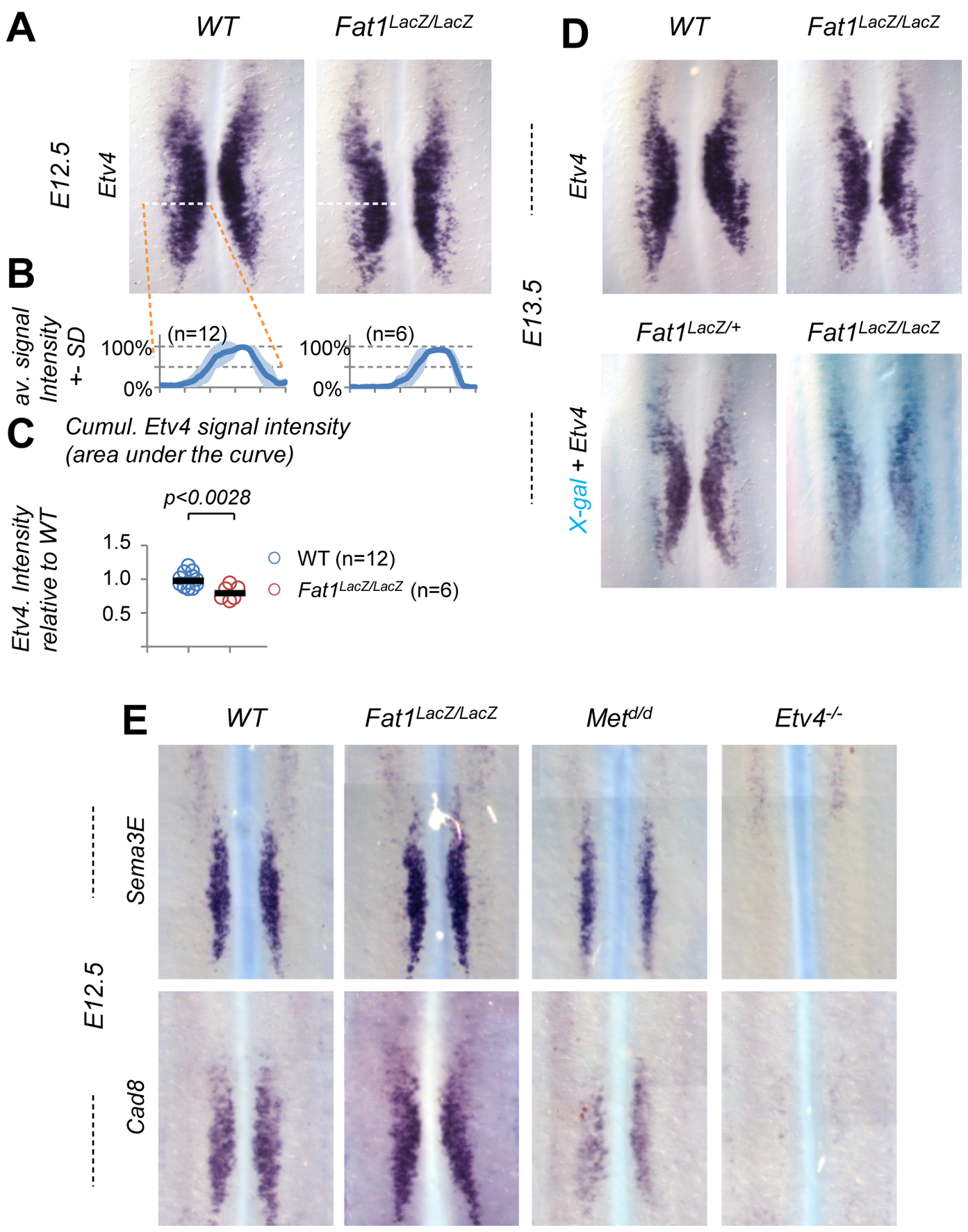

Supplement: S7 Fig — (A–C) (A) Etv4 expression was analyzed by ISH in E12.5 wild-type and Fat1LacZ/LacZ spinal cords. The images represent flat-mounted spinal cords in the brachial region. (B) Quantifications of Etv4 signal: each plot represents the average signal distribution (± standard deviation in light blue) measured on the indicated number of spinal cord sides along the orange dotted line in each image in (A) (Fat1+/+ [n = 12]; this set of controls is the same as that shown in Fig 6); Fat1LacZ/LacZ (n = 6). (C) Quantifications and statistical analyses of the sum of signal intensity corresponding to the area under the curves in plots shown in (C): each dot represents the sum of Etv4 intensity for each spinal cord side, the number of samples being indicated (the two sides of each embryo considered independent). (B–C): Underlying data are provided in S1 Data. (D) Analysis of Etv4 expression was carried out by ISH with Etv4 probe alone (top) on WT and Fat1LacZ/LacZ E13.5 spinal cords, or with Etv4 probe combined with prior X-gal staining (bottom) on Fat1LacZ/+ and Fat1LacZ/LacZ E13.5 spinal cords. (E) Analysis by ISH of Sema3E and Cadherin8 expression in flat-mounted brachial spinal cords from wild-type, Fat1LacZ/LacZ, Metd/d, and Etv4-/- embryos at E12.5: expression of Sema3E and Cadherin8 in the C7–C8 segments is lost in Etv4-/- and reduced in Metd/d mutants, whereas they are unaffected in Fat1LacZ/LacZ spinal cords. Cad8, Cadherin 8, Etv4, Ets variant gene 4; ISH, in situ hybridization; Sema3E, semaphoring 3E; WT, wild-type; X-gal, substrate for β-galactosidase activity. (TIF) [file pbio.2004734.s010.tif]

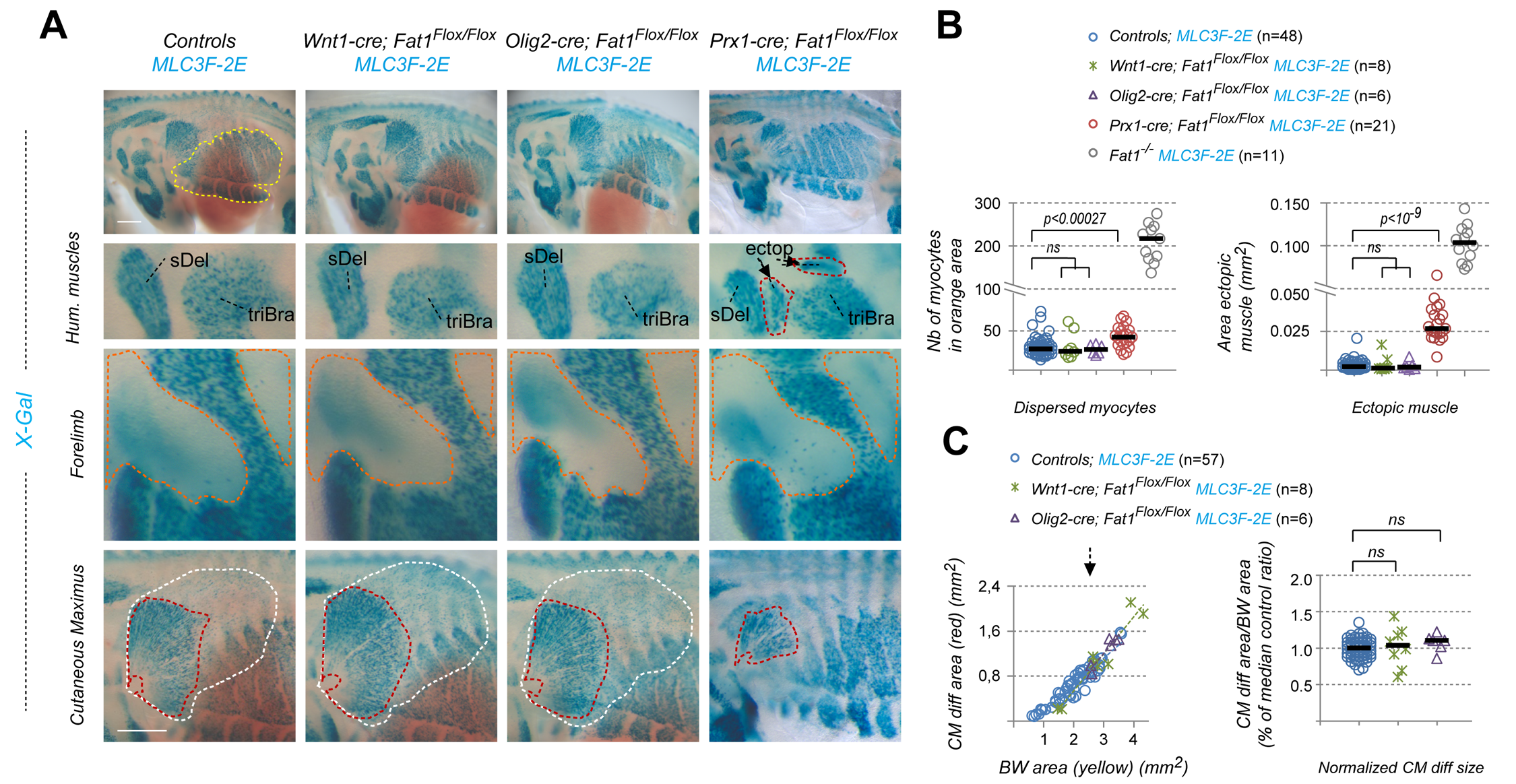

Supplement: S8 Fig — (A) Embryonic musculature was visualized by X-gal staining on E12.5 embryos carrying the MLC3F-2E transgene in different contexts of tissue-specific deletion of Fat1, with Wnt1-cre (neural crest cells), Olig2-cre (MN precursors), and Prx1-cre (limb/lateral mesoderm-derived mesenchyme) (cre lines described in S1 Table). Top images are images of the entire flank of Fat1Flox/Flox; MLC3F-2E controls, and Wnt1-cre; Fat1Flox/Flox; MLC3F-2E, Olig-cre; Fat1Flox/Flox; MLC3F-2E, and Prx1-cre; Fat1Flox/Flox; MLC3F-2E embryos, respectively. Higher magnification pictures focus (from top to bottom) on humeral muscles, on the forelimb area, and on the flank region in which the CM spreads, respectively. (B) Quantification of the number of dispersed myocytes found in orange areas in the forelimb (left plot) and of the area occupied by the ectopic humeral muscle (ectop) appearing between the spinodeltoid (Del) and the triceps brachii (TriBra) muscles (right plot). In both graphs, data from Fat1-/- embryos (from plots in S1B Fig) have been added in the last lane, for comparison of effect size. Note that on both graphs, there is an interruption on the y axis and a change of scale. Only Prx1-cre; Fat1Flox/Flox; MLC3F-2E embryos exhibit a mild increase compared to controls. Underlying data are provided in S1 Data. (C) Quantification of the expansion rate of differentiated CM fibers in Fat1Flox/Flox; MLC3F-2E controls, Wnt1-cre; Fat1Flox/Flox; MLC3F-2E, and Olig-cre; Fat1Flox/Flox; MLC3F-2E. Quantifications of Prx1-cre; Fat1Flox/Flox; MLC3F-2E embryos are shown in Fig 7B. Left graph: for each embryo side, the area covered by differentiated CM fibers was plotted relative to the area occupied by body wall muscles. Right plot: for each embryo, the CM area/body wall area was normalized to the median ratio of control embryos. Blue dots: Fat1Flox/Flox; MLC3F-2E (n = 57, includes the same set of controls as Fig 7B); green crosses: Wnt1-cre; Fat1Flox/Flox-; MLC3F-2E (n = 8); purple triangles: Oli [file pbio.2004734.s011.tif]

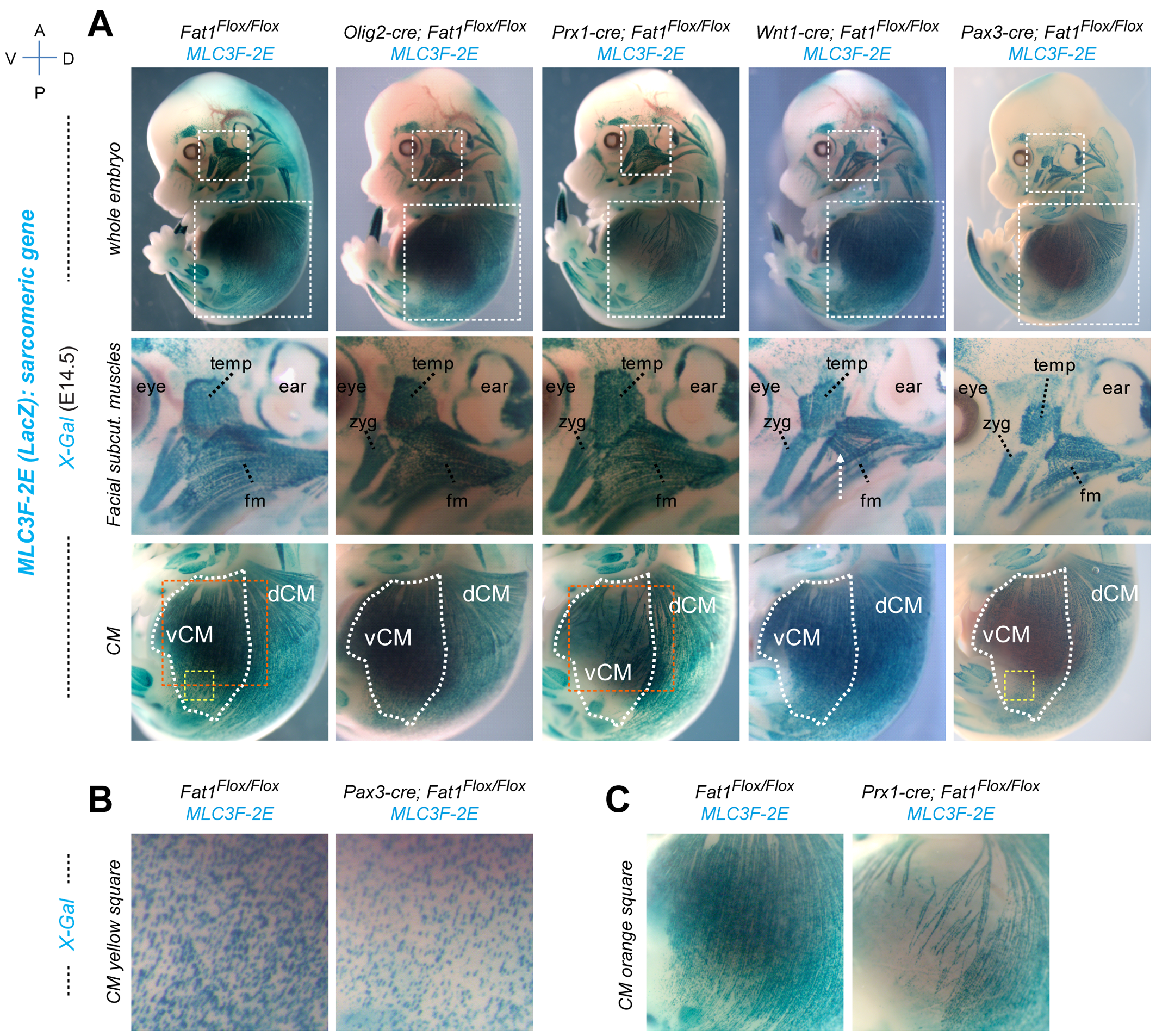

Supplement: S9 Fig — (A) The respective effects of depletion with Wnt1-cre, Olig2-cre, Prx1-cre, and Pax3-cre (premigratory myogenic cells + neural crest lineage) (cre lines described in S1 Table) were assessed by whole-mount X-gal staining on E14.5 Fat1Flox/Flox; MLC3F-2E controls (first column), Olig-cre; Fat1Flox/Flox; MLC3F-2E (second column), Prx1-cre; Fat1Flox/Flox; MLC3F-2E (third column), Wnt1-cre; Fat1Flox/Flox; MLC3F-2E (fourth column), and Pax3cre/+; Fat1Flox/ Flox; MLC3F-2E (fifth column) embryos. Top panels: low-magnification pictures show whole-embryo side views. Boxed areas show the position of higher magnification pictures shown in lower panels. Middle panels focus on facial subcutaneous muscles. Lower panels focus on the CM muscle. Prx1-cre; Fat1Flox/Flox embryos display normal musculature in the face but abnormal muscle shape and intramuscular orientation in the CM (higher magnification of the orange box shown in [C]), while Wnt1-cre; Fat1Flox/Flox embryos have a normal CM but severely affected subcutaneous muscles in the face, compared to controls (Fat1Flox/Flox). Thus, ablation in neural crest cells causes severe alterations in shape of the facial subcutaneous muscles in both Pax3cre/+; Fat1Flox/Flox and Wnt1-cre; Fat1Flox/Flox embryos. In contrasts, muscle shapes of Olig2-cre; Fat1Flox/Flox embryos are indistinguishable from those seen in controls. (B, C) Higher magnification views of fiber density in the CM in yellow and orange boxed areas outlined in lower panels in (A), respectively comparing control with Pax3cre/+; Fat1Flox/Flox (B) and with Prx1-cre; Fat1Flox/Flox (C) embryos. The effect of Fat1 ablation in Pax3cre/+; Fat1Flox/Flox embryos on CM appearance (B) is modest at E14.5 (high-magnification images illustrate a subtle reduction in the density of LacZ-positive nuclei). In contrast, the reduction in fiber density observed in Prx1-cre; Fat1Flox/Flox embryos (C) is very severe in the ventral CM, in contrast to more dorsal regions, coinciding with the limit a [file pbio.2004734.s012.tif]

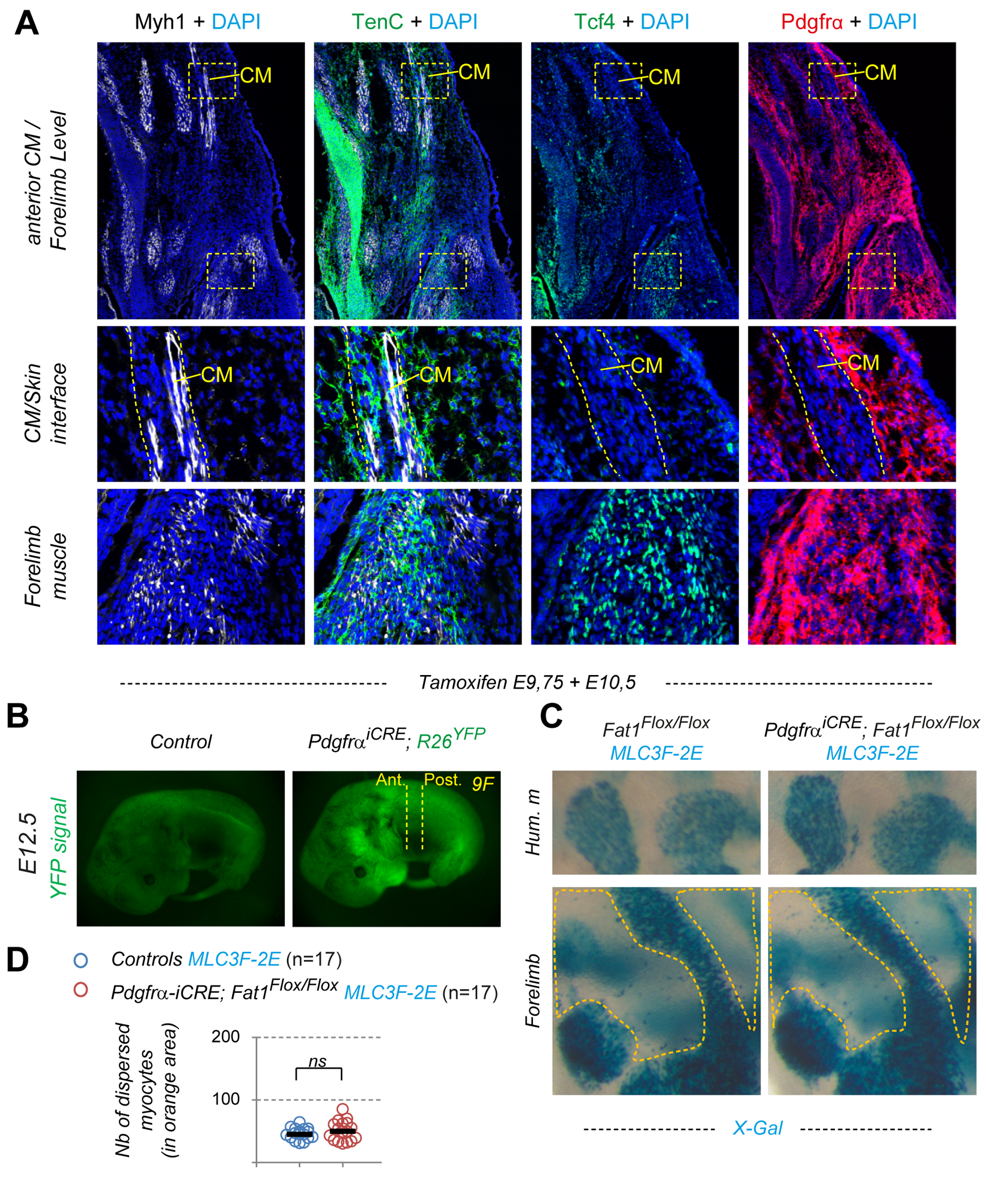

Supplement: S10 Fig — (A) Characterization of the mesenchyme subtype at the CM–skin interface. Sections of an E12.5 wild-type embryo at the level of the anterior CM (and upper forelimb) were immunostained with antibodies against Myh1 (white), TenascinC (green), Tcf4 (green), Pdgfrα (red), and DAPI (blue). The lower panels represent higher magnifications of the two bowed areas indicated in the low magnification views, focusing on the mesenchyme surrounding the CM and at the interface between CM and skin (middle panels) and the extremity of one forelimb muscle (lower panel). Whereas Tcf4 is detected in myogenic cells and connective tissue fibroblasts associated with the forelimb muscle, no expression is detected in the CM/skin connective tissue, which, in contrast, expresses TenascinC and Pdgfrα. (B) Control and Pdgfrα-iCre; R26YFP/+ embryos were collected at E12.5 from a pregnant female treated with tamoxifen at E9.5 + E10.5. Images show the direct YFP fluorescence signal acquired with the same exposure time. The vertical lines in the lower picture highlight the level of sections corresponding to Fig 9F (anterior CM and posterior CM). (C) Whole-mount β-galactosidase staining was performed on tamoxifen-treated control; MLC3F-2E (left) and Pdgfrα-iCre; Fat1Flox/Flox- ; MLC3F-2E (right) embryos at E12.5. Top images represent the upper forelimb region (with scapulohumeral muscles); bottom images represent the forelimb region, where myocyte dispersal is being quantified in (D). (D) Quantification of the number of dispersed myoblasts observed in the forelimb (counted in the areas delimited by orange lines). Blue dots: Controls; MLC3F-2E (n = 17, control embryos from tamoxifen-treated litters); red dots: Pdgfrα-iCre; Fat1Flox/Flox- ; MLC3F-2E (n = 17). Underlying data are provided in S1 Data. CM, cutaneous maximus; iCre, short form of CRE/ERT2; Myh1, myosin heavy chain I; Pdgfrα, platelet-derived growth factor receptor alpha; R26, Rosa26 locus; Tcf4, T cell factor 4; TenC, Tenascin C. (TIF) [file pbio.2004734.s013.tif]

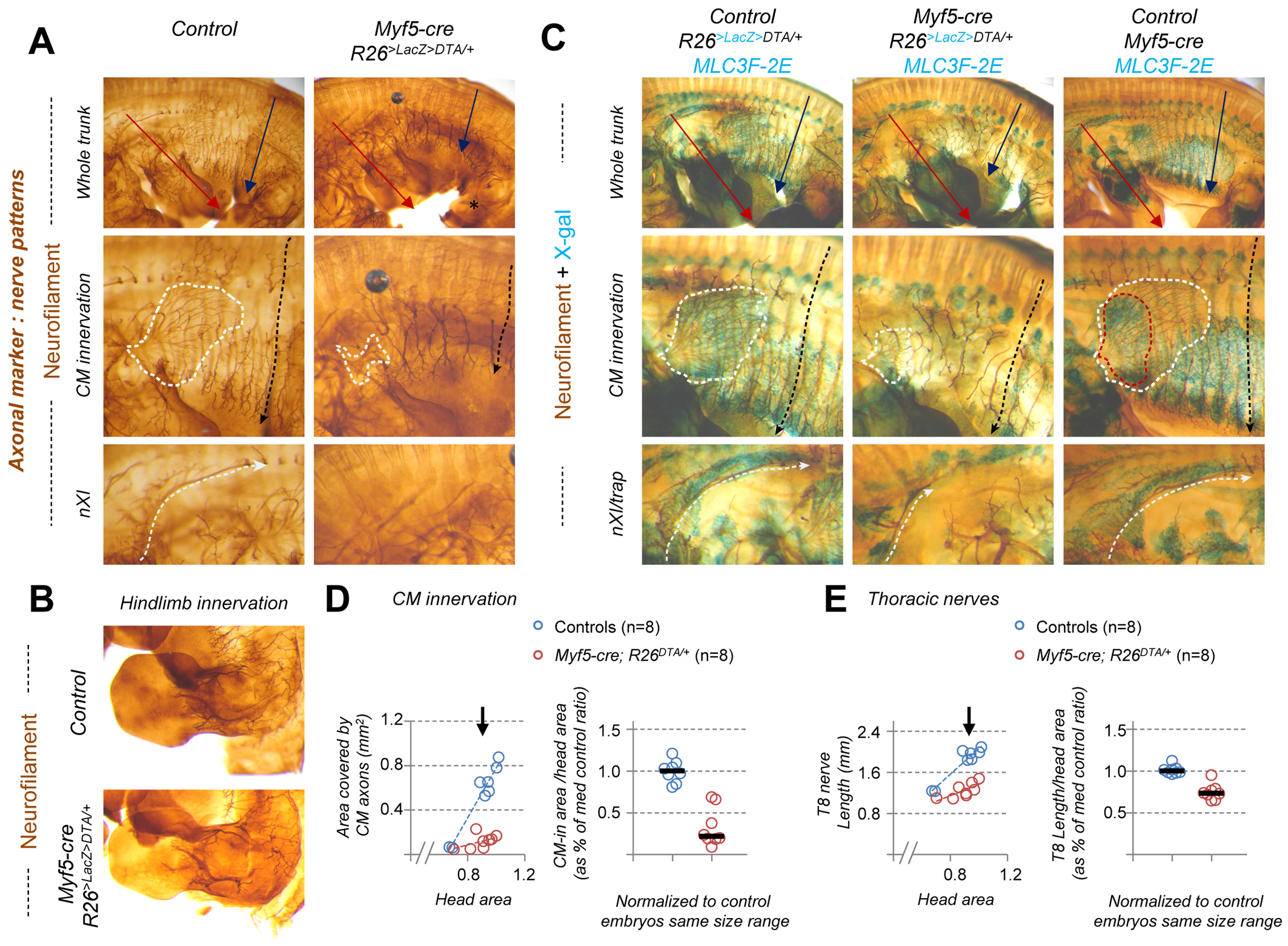

Supplement: S11 Fig — (A–C) Anti-neurofilament IHC was performed on E12.5 embryos, in the context of Myf5-cre-driven (S1 Table) myogenic lineage depletion, mediated by conditional DTA expression under regulatory control of the R26 locus (R26Lox-LacZ-STOP-Lox-DTA mouse line S1 Table). This nerve pattern is either visualized on its own (A, B) or combined with X-gal staining, when embryos also carry the MLC3F-2E-LacZ transgene (C). In cases with MLC3F-2E (C), X-gal staining also detects LacZ expression driven by the R26 locus in unrecombined cells (the relative expression levels in the two loci [R26 and MLC3F-2E-LacZ transgene] allows visualizing muscles in the trunk area but not in the limb). After BB-BA clearing, the embryos were cut in half and the internal organs and skin removed to visualize the CM and brachial plexus. Genotypes shown are control R26Lox-LacZ-STOP-Lox-DTA; and Myf5-cre; R26Lox-LacZ-STOP-Lox-DTA embryos (A, B); control R26Lox-LacZ-STOP-Lox-DTA; MLC3F-2E (left), Myf5-cre; R26Lox-LacZ-STOP-Lox-DTA; MLC3F-2E (middle), or Myf5-cre; R26Lox-LacZ-STOP-Lox-DTA; MLC3F-2E (right) (C). Upper panels show the entire flank of embryos at comparable stages. The area covered by CM-innervating axons is outlined with white dotted lines. The extent of the T8 thoracic nerve is outlined with a black arrow. The length of the forelimb (slightly affected by muscle depletion) is highlighted by a red arrow. Middle panels show higher magnification of the upper thoracic region containing the CM and corresponding motor axons as well as thoracic nerves, after manual removal by dissection of most cutaneous sensory nerves. Lower panels show the upper cervical region, with the trapezius muscle, innervated by the cranial nerve XI, also called spinal accessory nerve. (D, E) Quantifications of the progression of CM innervation (D) and of thoracic nerve growth (E). Left plots: for each embryo side, the area covered by CM-innervating axons (D) or the length of the T8 nerve (E) were plotted relative to the hea [file pbio.2004734.s014.tif]

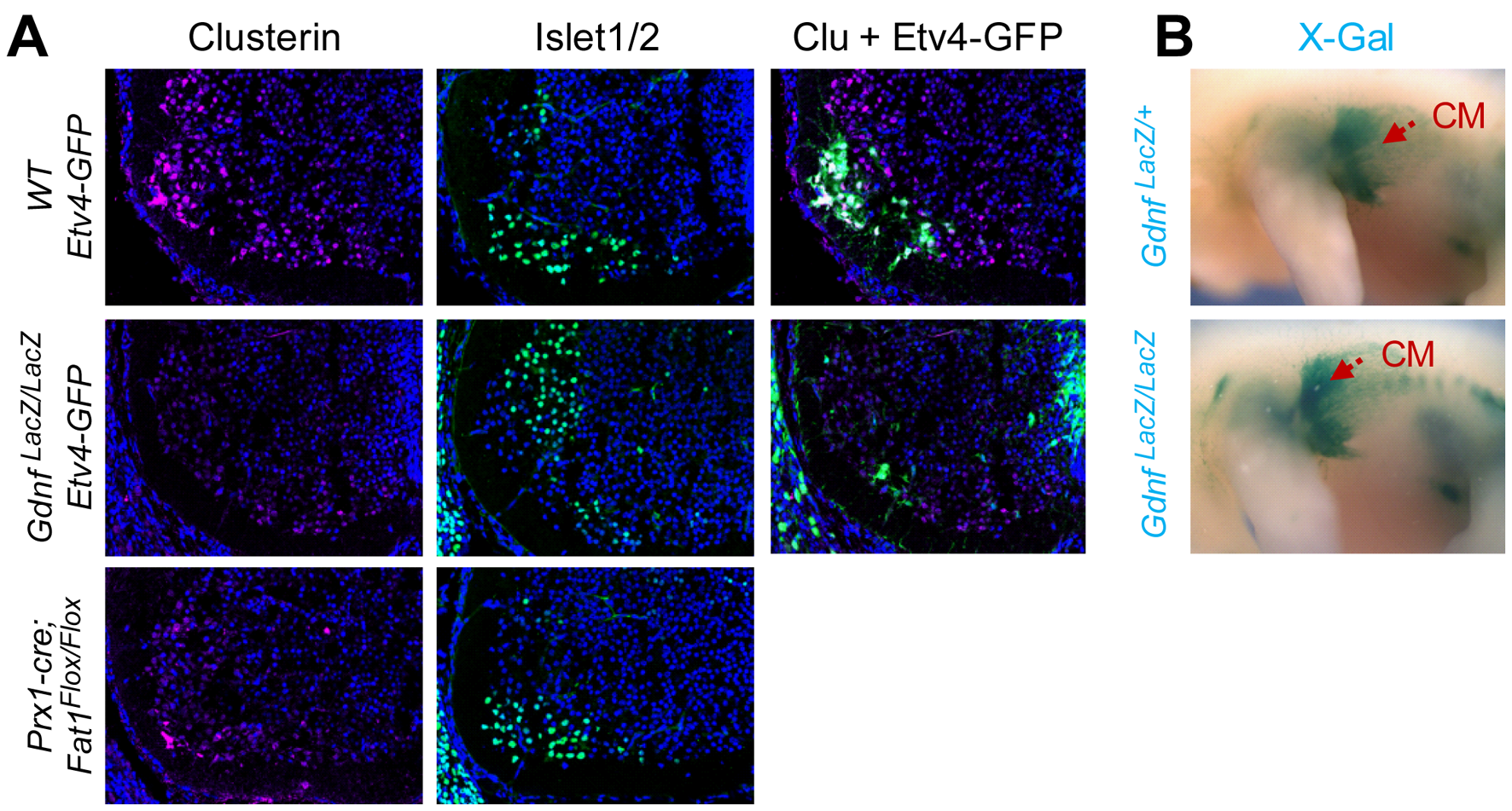

Supplement: S12 Fig — (A) Analysis by IHC of Clusterin, islet1, and GFP expression on spinal cord sections at the C7–C8 level from E13.5 WT; Etv4-GFP/+, from GdnfLacZ/LacZ; Etv4-GFP/+, and from Prx1-cre; Fat1Flox/Flox embryos. Clusterin and GFP staining were done on sections neighboring those analyzed with Islet1/2 IHC. Etv4-GFP could not be combined with Prx1-cre, as this combination appears to cause a phenotype (short limbs, with impaired elongation, visible from E12.5 onward) unrelated to Fat1 alleles and most likely linked to the site of integration of both transgenes. (B) X-gal staining of GdnfLacZ/+ and GdnfLacZ/LacZ embryos, showing that expansion of the area covered by GdnfLacZ-expressing CM progenitors is not affected by the lack of Gdnf. CM, cutaneous maximus; cre, cre recombinase; Etv4, Ets variant gene 4; GFP, green fluorescent protein; IHC, immunohistochemistry; MN, motor neuron; WT, wild-type; X-gal, substrate for β-galactosidase activity. (TIF) [file pbio.2004734.s015.tif]
